# Walking and balance outcomes for stroke survivors: a randomized clinical trial comparing body-weight-supported treadmill training with versus without challenging mobility skills

**DOI:** 10.1186/s12984-018-0442-3

**Published:** 2018-11-01

**Authors:** Sarah A. Graham, Elliot J. Roth, David A. Brown

**Affiliations:** 1 Departments of Physical and Occupational Therapy, University of Alabama at Birmingham, Building 516 20th Street South, Birmingham, AL 35233-4555 USA; 2 Department of Physical Medicine and Rehabilitation, Feinberg School of Medicine, Northwestern University, Chicago, IL USA

**Keywords:** Poststroke rehabilitation, Walking, Balance, Body-weight-support treadmill training, Robotics, Hemiparesis, Mobility skills

## Abstract

**Background:**

Treadmill training, with or without body-weight support (BWSTT), typically involves high step count, faster walking speed, and higher heart-rate intensity than overground walking training. The addition of challenging mobility skill practice may offer increased opportunities to improve walking and balance skills. Here we compare walking and balance outcomes of chronic stroke survivors performing BWSTT with BWSTT including challenging mobility skills.

**Methods:**

Single-blind randomized clinical trial comparing two BWSTT interventions performed in a rehabilitation research laboratory facility over 6 weeks. Participants were 18+ years of age with chronic (≥5 months) poststroke hemiparesis due to a cortical or subcortical ischemic or hemorrhagic stroke and walking speeds < 1.1 m/s at baseline. A hands-free group (HF; *n* = 15) performed BWSTT without assistance from handrails or assistive devices, and a hands-free plus challenge group (HF + C; *n* = 14) performed the same protocol while additionally practicing challenging mobility skills. The primary outcome was change in comfortable walking speed (CWS), with secondary outcomes of fast walk speed (FWS), six-minute walk distance, Berg Balance Scale (BBS) scores, and Activities Specific Balance Confidence (ABC) scores.

**Results:**

Significant pre-post improvement of CWS (*Z = − 4.2, p ≤ 0.0001*) from a median of 0.35 m/s (range 0.10 to 1.09) to a median of 0.54 m/s (range 0.1 to 1.17), but no difference observed between groups (*U = 96.0, p = 0.69*). Pre-post improvements across all participants resulted in reclassified baseline ambulation status from sixteen to ten household ambulators, three to seven limited community ambulators, and ten to twelve community ambulators. Secondary outcomes showed similar pre-post improvements with no between-group differences.

**Conclusions:**

The addition of challenging mobility skills to a hands-free BWSTT protocol did not lead to greater improvements in CWS following 6 weeks of training. One reason for lack of group differences may be that both groups were adequately challenged by walking in an active, self-driven treadmill environment without use of handrails or assistive devices.

**Trial registration:**

NCT02787759 Falls-based Training for Walking Post-Stroke (FBT); retrospectively registered June 1st, 2016.

## Introduction

A major focus of stroke rehabilitation is to improve walking function to a safe level for community ambulation. In particular, body-weight-supported treadmill training (BWSTT) is a technique that yields moderate improvements in walking function for individuals in both subacute and chronic phases poststroke [1–5]. The most recent Cochrane Review [6] determined that BWSTT increased walking velocity (i.e., 0.06 m/s on the 10-m walk test) and endurance (i.e., 14.19 m on the six-minute walk test); however, it did not improve walking function to a greater extent than other interventions. One essential requirement for functional walking is successful navigation of everyday walking challenges, which require mobility skills that allow a person to avoid, or recover from, balance disruptions [7, 8]. However, few BWSTT protocols include chances to navigate walking challenges (e.g., obstacles, distractions, etc.). Protocols that do include challenges generally incorporate them during an overground training component. For example, the LEAPS trial [9] and other smaller studies [3, 10, 11] incorporated overground mobility skills into their protocols and saw large improvements in walking speed (i.e., > 0.2 m/s).

Mobility skill practice may be essential for walking improvements; however, combining BWSTT with overground training makes it difficult to discern whether improvements in walking speed should be attributed to the BWSTT or overground component. BWSTT protocols also commonly allow handrail use and involve manual assistance from a therapist or robot [9, 10, 12, 13] further making it difficult to determine which components of a training protocol are critical for improvements. Thus, we investigated the efficacy of challenging skill practice during walking rehabilitation by incorporating it into one of two comparable protocols and further isolated the effects of skill practice through *eliminating use of handrails or assistance from both protocols*. We focused solely on a treadmill paradigm since it offers advantages over training overground for such challenging skill practice including safety, minimal space requirements, and the ability to repetitively perform a high number of skill repetitions without interruption [14, 15].

The purpose of this single-blind, randomized controlled clinical trial was to compare walking and balance outcomes of two BWSTT protocols over a six-week period. A hands-free (HF) protocol involved only BWSTT and a hands-free plus challenge (HF + C) protocol was equivalent except for the addition of challenging mobility skills. The selected skills encompassed seven of eight dimensions of community mobility proposed by Patla and Shumway-Cook [7, 8]. We expected both groups to improve but expected that performing challenging mobility skills would improve overground CWS for the HF + C group to a greater extent than the HF group. We also expected that improvements would be greater for the HF + C group after a six-month follow-up period. We expected these findings because HF + C training offered opportunities for participants to develop new and improved balance and gait-control strategies and increased confidence in responding to balance disturbances, as compared with HF walking. We secondarily explored changes in fast walk speeds (FWS), six-minute walk distance, Berg Balance Scale (BBS) scores, and Activities-Specific Balance Confidence (ABC) scores.

## Methods

This study was a single-blind randomized clinical trial comparing walking and balance outcomes of two BWSTT interventions, HF versus HF + C, performed in a rehabilitation research laboratory facility over 6 weeks. We selected walking and balance outcomes of CWS, FWS, BBS, and ABC scores due to their strong clinical relevance, association with ambulation status after stroke, and expert panel recommendations [16–20].

### Power calculations

We performed a power calculation for the primary outcome variable, change in CWS, based on a repeated measures ANCOVA (covariate baseline walking speed) with two groups, *p* ≤ 0.05 to reject the null hypothesis, 80% power, and 0.5 effect size to detect a gait velocity difference of at least 0.16 m/s - the proposed minimal clinically important difference [21]. Given these parameters the estimated sample size was 16 individuals per group.

### Participants

**Fig. 1:**
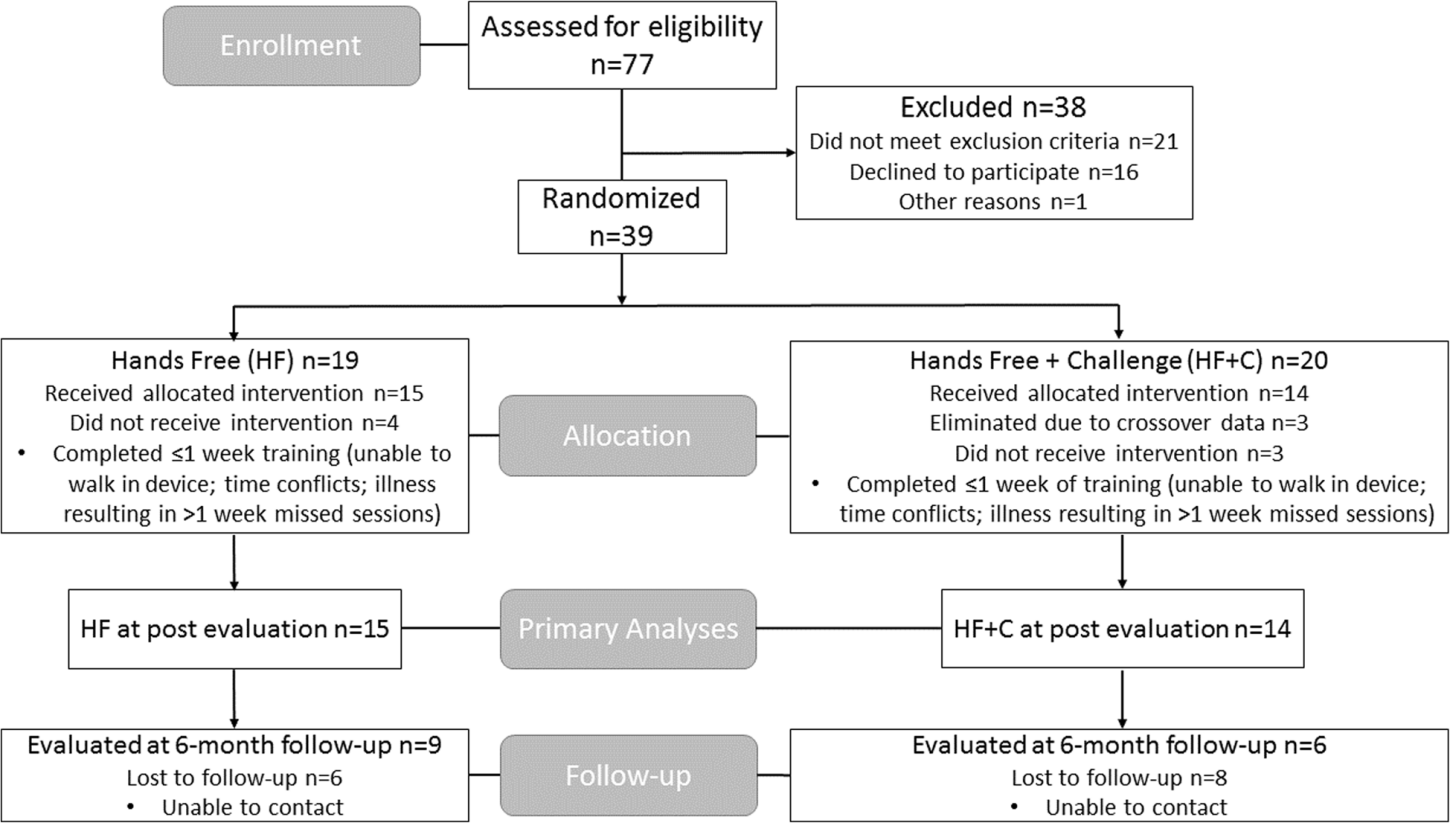
Consort diagram for participant flow through study

We recruited participants from June 2012 through January 2015 until the end of our funding period and we completed data collection through follow-up in January 2016. We randomized 39 individuals over 18 years of age with chronic (≥5 months) poststroke hemiparesis due to cortical or subcortical ischemic or hemorrhagic stroke confirmed by computed tomography, magnetic resonance imaging, or clinical criteria (Fig. 1). If participants were unable to complete the first week of training we removed them from subsequent analyses, because they had not experienced more than three sessions of the training protocol. This decision yielded *N* = 29 individuals for our primary analysis; *n* = 15 in the HF and *n* = 14 in the HF + C group. We will refer to these numbers from this point on throughout the text.

Participants were medically approved for exercise, able to ambulate ≥14 m with or without an assistive and/or orthotic device but had slow CWS < 1.1 m/s at baseline. Exclusion criteria were serious medical conditions; resting systolic blood pressure > 180 mmHg and/or diastolic blood pressure > 110 mmHg; resting heart rate > 100 bpm; spasticity management including botulinum toxin injection (< 4 months) or phenol block (< 12 months) to the affected lower extremity; intrathecal or oral baclofen (< 30 days); Mini-Mental State Exam score < 24; currently undergoing lower-limb physical therapy; participation (< 6 months) in long-term (> 4 weeks) BWSTT, limb-loaded pedaling, or lower-extremity strengthening; plans to move out of area; and transportation barriers to study site.

### Assessments

All participants gave informed consent as approved by the Institutional Review Board of the University of Alabama at Birmingham prior to initial assessment (protocol #: F120425008). We contacted a participant’s physician regarding any concerns raised to determine appropriateness for study participation and to clarify any specific exercise precautions.

It was not practical for research staff overseeing training to be blinded to intervention group, but a blinded physical therapist conducted all assessments. The physical therapist assessed outcomes for CWS, FWS, six-minute walk distance, BBS, and ABC scores at baseline, mid study (i.e., 3 weeks; all except ABC), immediately post intervention, and six-months post intervention. Participants performed the 10-m walk in a straight hallway with no obstructions and instructions to “walk at the speed that feels most comfortable to you” for CWS and “walk at the fastest speed that you feel you can safely attain” for FWS. We determined CWS as the average of three trials and FWS as the fastest time achieved out of three attempts, with sufficient rest provided as necessary between trials. Participants performed the six-minute walk around an oval 85-ft walkway while the therapist followed with a Stanley distance wheel to record distance covered. We allowed participants to use an assistive device and/or ankle-foot orthosis as necessary during assessments. We considered the following changes in outcome measures to be clinically meaningful: 10-m walk 0.16 m/s; [21] six-minute distance 34.4 m; [22] BBS 4.13 points; [23] and ABC increase above 67% (fall-risk threshold) [24]. We used the Stroke Impact Scale (SIS), Dynamic Gait Index (DGI), and Geriatric Depression Scale (GDS) to further characterize participants.

### Randomization to study groups

We stratified randomization by baseline CWS (severe < 0.5 m/s; moderate ≥0.5 m/s). We used a random number generator (www.random.org) to generate two lists of “0” and “1” sequences, one for each stratification. As each sequential participant entered the study, we allocated the next “0” or “1” value to that person. We designated “0” for the HF group and “1” for the HF + C group. The lab coordinator sequentially enrolled participants and the principal investigator assigned qualified participants to groups prior to initiating training. Using this procedure, we successfully achieved balanced groups on baseline walking behaviors (speed classification, use of overground assistive devices, orthoses, etc. (Table 1)). We scheduled training sessions such that one participant performed training at a time, minimizing exposure between groups. We did not inform participants about the a priori expectation of greater walking improvements for participants who performed mobility skills during training.

**Table 1:** Classification of baseline walking measures for each group

Walking Behavior	Hand Free *n* = 15	Hands Free + Challenge *n* = 14	*p* value
Walking speed severity (severe < 0.5; moderate≥0.5)	7 moderate 8 severe	6 moderate 8 severe	0.84
Community Walking Status	8 HH; 2 LC; 5 C	8 HH; 1 LC; 5 C	0.86
Assistive Devices used for overground assessments	*n* = 6 none *n* = 2 single-point cane *n* = 5 quad cane *n* = 1 hemi walker *n* = 1 other	*n* = 5 none *n* = 5 single-point cane *n* = 2 quad cane *n* = 2 hemi walker	0.41
Ankle-Foot Orthosis	*n* = 6 none *n* = 5 rigid plastic, no joint *n* = 3 rigid plastic, joint *n* = 1 external ankle support	*n* = 11 none *n* = 2 rigid plastic, joint *n* = 1 metal double upright with joint	0.07
Functional Ambulation Classification	*n* = 3 Dependent, Level II *n* = 3 Independent, level surfaces only *n* = 9 Independent, level and non-level surfaces	*n* = 1, Dependent, supervision *n* = 1, Independent, level surfaces only *n* = 12, Independent, level and non-level surfaces	0.14

*p values for chi square comparisons between groups; household (HH); limited community (LC); community (C))

### Training environment

All walking training occurred at the University of Alabama at Birmingham Locomotor Control and Rehabilitation Robotics Laboratory over a Bertec treadmill while supported by a self-driven robotic device called the KineAssist [25, 26]. The KineAssist provided different levels of BWS at the approximate body center of mass through a hip/pelvis interface and maintained prescribed BWS during all phases of the gait cycle. The KineAssist operates via “cobotics”, which is software that senses human movement and allows devices to take direction from this movement. Participants interacted with the KineAssist via a pelvic mechanism equipped with force transducers that sense forces being applied by the participant. The cobotics software sent these forces to the treadmill control panel, which in turn commanded the treadmill belts to run at a given speed based on a predetermined force-velocity relationship.

In this manner, the KineAssist allowed “self-driven” or intentional movement in six degrees of freedom, including translational movement in three perpendicular axes: surge (forward/backward movement over the treadmill), heave (vertical center of mass motion), and sway (side-to-side translation); and rotational movement about three perpendicular axes: roll (hip hiking), pitch (forward/backward tilting), and yaw (left/right rotation). A torso harness provided additional support of upright orientation by preventing extreme forward lean in the event of a loss of balance. Both training groups walked without the use of handrails or assistive devices, which were unnecessary given the KineAssist’s safety mechanisms. When participants lost their balance, the device caught them after a short descent and the clinician/researcher assisted them into a standing position to continue training with little interruption. We also discouraged participants from using the clinician/researcher for support.

### Intervention protocols

Intervention protocols adhered to physical activity recommendations (i.e., 20–60 min of aerobic exercise ≥3 days/week) of the American Heart Association for stroke survivors [27]. Interventions comprised 18 sessions of 30-min walking over 6 weeks. Participants received alternate rest days to minimize excessive fatigue (training Monday, Wednesday, and Friday). We rescheduled missed sessions to occur on one of the two free days in the weekly schedule.

Two trained research staff oversaw each training session. Sessions began with participants performing a BWS test over the treadmill that involved timed 10-m walks at three or four levels of BWS (0, 10, 20, and/or 30%) depending on height constraints and/or comfort with higher BWS. We selected the lowest BWS per session that resulted in the participant walking ≥0.08 m/s (minimal detectable change) [21] faster than with 0% support. In some cases 0% BWS facilitated the fastest training speed. This approach differed from other BWSTT protocols that generally started training with high BWS (30–40%) and progressed to lower levels [1, 5, 9, 10, 13]. However, the approach used in this study allowed participants to train with BWS that best facilitated their fastest CWS on a session-by-session basis.

We asked all participants to walk at a target intensity of 60–80% heart rate reserve based on the Karvonen formula [28]. We calculated max heart rate as 220 – age and then used the Karvonen formula (target heart rate = ((max heart rate − resting heart rate) × % intensity) + resting heart rate) to determine the training range for each participant. We used this target heart rate range in order to ensure comparable intensity between training groups. We monitored heart rate and Borg Scale rating of perceived exertion (RPE) each minute (heart rate) and every other minute (RPE) to document participants’ actual and perceived exertion, respectively. Participants walked with their selected BWS at a speed that maintained their heart rate within the target range for six, five-minute bouts. We required participants to take brief standing breaks if their heart rate exceeded 80% heart rate reserve and allowed them to take voluntary breaks if necessary; however, encouraged them to continue walking as soon as possible. The clock continued running for both heart rate and voluntary breaks. Every 5 minutes we offered participants a seated break; however, as participants progressed with training we encouraged them to walk continuously and some individuals attained 30 continuous minutes of walking by the end of 6 weeks. We additionally tracked number of steps with a step watch (Orthocare Innovations) worn on the nonparetic limb and distance traveled with a Stanley distance wheel placed over the edge of the treadmill.

During each *HF + C protocol session*, participants walked for the same 30-min training period but in addition to walking performed three of nine mobility skills for 10 min per skill (Table 2) that we randomized such that participants practiced all nine skills over three successive sessions. We adjusted the level of challenge for each participant to allow for failed attempts. These challenging mobility skills encouraged participants to learn how to avoid losing balance and to adapt to changing conditions. All skills encompassed Patla & Shumway-Cook’s proposed mobility dimension of minimum walking distance, [7, 8] as participants were encouraged to take as many steps as possible while performing each skill. We did not include one of the dimensions (i.e., traffic considerations) for practical reasons related to the training environment. We have provided the other dimensions of mobility addressed by each skill in Table 2.

**Table 2:** Nine walking skills experienced by participants in the HF + C group

Task	Delivery mechanism	Mobility dimension
Hurdles	A pliable hurdle positioned in front of participant’s paretic & nonparetic limb for 5 min each; challenge increased through raising hurdle height	terrain characteristics
Foam Shoes	3- or 6-in. foam blocks strapped to the bottom of participants’ shoes; challenge increased by using thicker foam or walking faster	terrain characteristics
Backward Walking	Participants walked backward; challenge increased by faster speeds	postural transitions
Head Turns	Participants asked to look in 4 different directions (i.e., up, down, left, or right) for 10s each; increased challenge through nodding head up/down & left/right for 10s each	attentional demands
Backward Perturbation	Participants exposed to sudden brief accelerations of the treadmill belt, of sufficient magnitude to disturb forward progression; challenge increased through larger accelerations	postural transitions; external physical loads
Speed Up/ Slow Down	Participants walked at double their CWS for 10s & then recovered at their CWS for 20s; challenge increased if CWS increased	time constraints; postural transitions
Variable Speeds	Participants walked at a variety of randomly selected speeds between 0.2 m/s < daily CWS and 0.2 m/s > daily CWS; challenge increased if CWS increased	postural transitions
Narrow Stepping	Participants walked in ambient lighting conditions while keeping their feet within a narrow area of the treadmill belt designated by laser light beams; challenge increased through narrowing lights	terrain characteristics; ambient lighting
Long Stepping	Participants walked in ambient lighting conditions while taking long steps with both feet over a laser beam placed in front of them; challenge increased by moving laser farther away	terrain characteristics; ambient lighting

### Statistical analyses

**Fig. 2:**
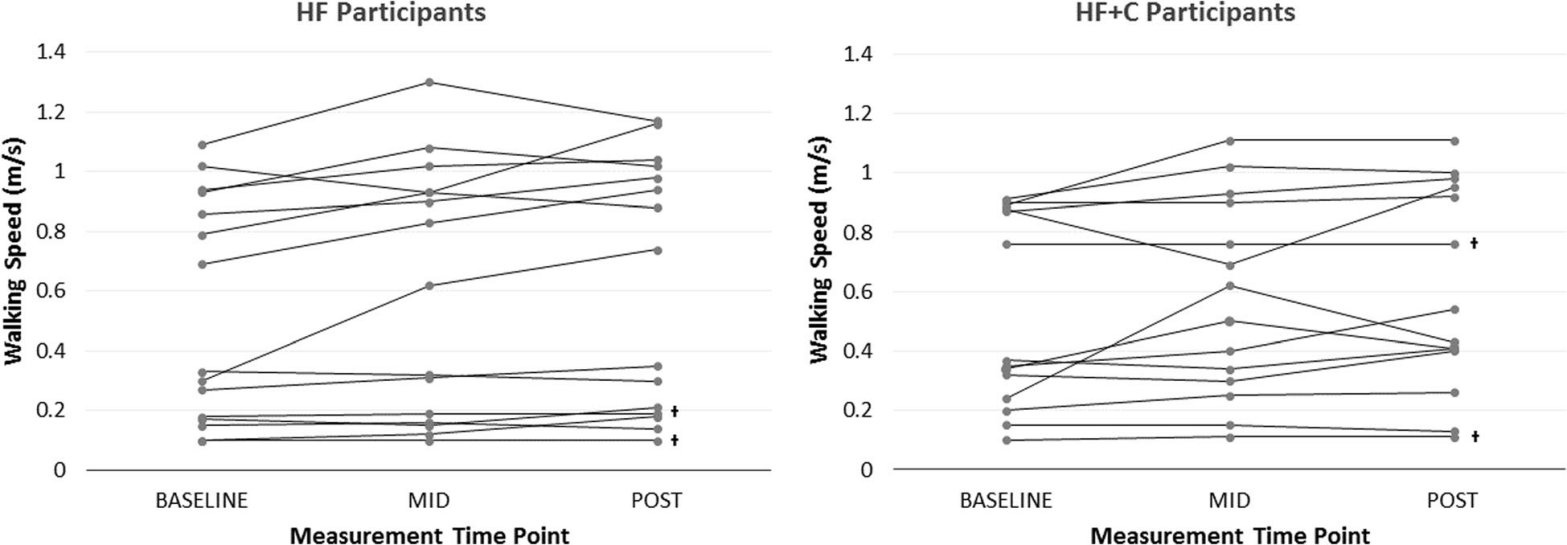
Baseline, mid, and post CWS measurements for *n* = 15 participants in the HF group (left) and *n* = 14 HF + C group (right). Participants with the midpoint measurement carried forward are marked with a † at post

We used SPSS version 24 to conduct all statistical tests and used *p* ≤ 0.05 to define statistical significance. We compared baseline characteristics with unpaired *t* tests (continuous variables) and chi square tests (categorical variables). We examined outcome data for completeness and discovered that 13.3% (*n* = 2) of our post measurement outcomes were missing for the HF group and 14.2% (*n* = 2) for the HF + C group. We therefore used a last measurement carried forward imputation approach and used the mid-assessment data point for these participants (noted in Fig. 2).

We next examined correlations between variables to determine if baseline CWS was significantly related to change in CWS. We did not find a significant relationship between these variables for either group (HF *r = 0.28, p = 0.45*; HF + C *r = 0.12, p = 0.57*); thus, we did not include baseline CWS as a covariate in analyses.

We conducted Shapiro-Wilks tests for each group for change in CWS and discovered a significant departure from normality for the HF group (*p = 0.004*); thus, we conducted a Mann-Whitney U test to determine if there were differences between groups. In the absence of group differences, we collapsed participants into a single group and checked pre and post CWS for normality. Both analyses revealed significant departures from normality (*p = 0.001* & *p = 0.004* respectively); thus, we conducted a Wilcoxon Signed Rank test to determine if participants significantly improved CWS pre to post assessment. We evaluated changes in community walking classification with a chi square analysis.

At six-month follow-up, we were missing 40% (*n* = 6) of CWS data for the HF group and 57% (*n* = 8) for the HF + C group. We therefore chose to analyze only complete data to determine if groups differed in CWS from post measurement to six-month follow-up. The complete data set was normally distributed (Shapiro Wilk *p > 0.05*); thus, we used a repeated measures ANOVA to test for main effects of group and time.

For exploratory analyses on our secondary outcome variables, we conducted a repeated measure MANOVA to determine if there were main effects of group or time (pre - post) for FWS, six-minute distance, BBS, or ABC scores.

## Results

### Characterization of participants at baseline

Although we randomly allocated participants to each intervention, groups significantly differed in age (HF + C was younger) and geriatric depression scale (HF exhibited greater depression) (Table 3). However, upon further analysis neither of these variables were significantly related to change in CWS for both groups. We found no significant differences between groups for any other measures.

**Table 3:** Participant baseline characteristics

Participant Characteristic	HF (*n* = 15)	HF + C (*n* = 14)	*p* value
Age (years), mean (SD)	60.3 (12.8)	48.9 (14.4)	0.03*
Gender, # males	7	8	0.57
Time since stroke (months), mean (SD)	47.7 (64.7)	52 (71.4)	0.87
Side of hemiparesis, # Left	9	11	0.28
Fugl-Meyer, mean (SD)	18.9 (5.1)	18.9 (6.5)	0.99
ABC (%), mean (SD)	56 (30)	73 (15)	0.09
GDS, mean (SD)	8.4 (6.7)	3.9 (3.5)	0.03*
DGI, mean (SD)	13.7 (6.0)	16.6 (4.5)	0.16
SIS mobility, mean (SD)	35.8 (8.5)	39.8 (4.4)	0.13
SIS ADL, mean (SD)	36.9 (10.3)	40.3 (6.2)	0.30
SIS participation, mean (SD)	28.8 (8.0)	30.4 (5.1)	0.52
CWS, mean (SD)	0.53 (0.38)	0.52 (0.32)	0.95
FWS, mean (SD)	0.71 (0.46)	0.77 (0.51)	0.76
6-min distance, mean (SD)	182.2 (131.9)	193.9 (113.7)	0.80
BBS, mean (SD)	42.5 (10.7)	45.9 (9.3)	0.37

*p ≤ 0.05

*ABC* Activities Specific Balance, *GDS* Geriatric Depression Scale, *DGI* Dynamic Gait Index, *SIS* Stroke Impact Scale, *CWS* Comfortable Walk Speed, *FWS* Fast Walk Speed, *BBS* Berg Balance Scale

Participants did not experience any adverse effects related to the training protocols. Results of the Mann-Whitney U test for change in CWS between groups revealed that groups were not significantly different (*U = 96.0, p = 0.69).* However, collapsed into a single group (*N* = 29) participants significantly improved (*Z = − 4.2, p ≤ 0.0001*) CWS pre to post assessment with an effect size *r = 0.55*, from a median of 0.35 m/s (range 0.10 to 1.09) to a median of 0.54 m/s (range 0.1 to 1.17), as indicated by the Wilcoxon signed-rank test (Fig. 2). Using the minimal clinically important difference of 0.16 m/s to classify participants as either “responders” or “nonresponders” as others have done, [10] there were five participants classified as responders (i.e., two HF and three HF + C).

Improvements in CWS resulted in reclassification of walking ability post intervention. At baseline, there were 16 participants with an ambulation level classified as household (< 0.4 m/s), three as limited community (0.4–0.8 m/s), and ten as community (> 0.8 m/s). Post intervention, these numbers changed significantly (*χ*^*2*^ *(4, N = 29) = 27.2, p < 0.0001)*, to ten household, seven limited community, and 12 community ambulators.

For participants with complete data (*n* = 9 HF; *n* = 6 HF + C), the repeated measures ANOVA revealed that groups did not significantly differ in CWS from post assessment to six-month follow-up (*p = 0.58*). We also did not detect a change in CWS from post to follow-up assessment (*p = 0.76*), with a HF group post (*M = 0.75 m/s; 95% CI 0.47 to 1.03)* vs. follow-up (*M = 0.78 m/s*; *95% CI 0.49 to 1.06)* and a HF + C group post (*M = 0.67 m/s*; *95% CI 0.33 to 1.01*) vs. follow-up (*M = 0.63 m/s; 95% CI 0.28 to 0.97*).

The exploratory repeated measure MANOVA for secondary outcomes (*n* = 15 HF; *n* = 14 HF + C) revealed similar findings to that of CWS. Although participants collectively improved performance in these outcomes pre to post assessment (Table 4), we did not detect a main effect of group for any variable (*p = 0.08 to 0.87*).

**Table 4:** Changes in secondary outcome measures pre to post assessment

Measure	Group	Pre Mean (95% CI)	Post Mean (95% CI)	Effect size *η*^2^	Sig. change pre to post
FWS (m/s)	HF _(*n* = 15)_	0.68 (0.42 to 0.93)	0.78 (0.49 to 1.07)	0.46	*p* ≤ 0.0001
	HF + C_(*n* = 14)_	0.77 (0.50 to 1.04)	0.88 (0.58 to 1.19)		
6-min distance (m)	HF _(*n* = 15)_	182.2 (116.8 to 247.6)	221.1 (146.6 to 295.5)	0.43	*p* ≤ 0.0001
	HF + C_(*n* = 14)_	193.9 (126.2 to 261.6)	225.6 (148.5 to 302.7)		
BBS (points)	HF _(*n* = 15)_	42.5 (37.2 to 47.9)	45.1 (39.6 to 50.6)	0.21	*p* = 0.01
	HF + C_(*n* = 14)_	45.9 (40.4 to 51.5)	47.6 (41.9 to 53.3)		
ABC (%)	HF _(*n* = 15)_	56 1 (43.7 to 68.6)	61.8 (51.6 to 72.0)	0.14	*p* = 0.05
	HF + C_(*n* = 14)_	71.9 (59.0 to 84.7)	74.3 (63.7 to 84.9)		

*FWS* fast walk speed, *BBS* Berg balance scale, *ABC* activities specific balance

## Discussion

We sought to determine whether HF + C BWSTT was superior compared to HF BWSTT in improving walking outcomes for individuals with chronic poststroke hemiparesis. Contrary to our primary hypothesis there were no differences in walking outcomes between groups. It is possible that the challenge of walking hands free was a strong enough stimulus to elicit walking and balance improvements regardless of group assignment. Nine of fifteen participants in the HF group and nine of fourteen in the HF + C group required assistive devices to perform overground walking assessments. However, we did not permit participants to use any form of assistive device during training. Thus, it is likely that walking without handrails over the self-driven treadmill sufficiently challenged dynamic balance of both HF + C and HF participants leading to improvements in overground walking outcomes.

Given that participants in both groups improved walking function, our findings demonstrated that stroke survivors can benefit from training involving challenging walking conditions. Recent recommendations for neurorehabilitation included training that involves many repetitions and continues to challenge the patient [29]. Training environments that incorporated skilled movements appeared to induce better and longer lasting improvements in motor function following cerebrovascular injury [29–36]. The nine mobility skills used in this study offered participants this experience; however, practicing these skills was not essential to engender improvements in walking function for these chronic stroke survivors.

While this finding could be considered surprising, both of our training protocols relied on the principle of active participant involvement. Winstein and Kay (2015) [37] authored an elegant review of learning-dependent neuroplasticity following neurological injury. They highlighted that repetitive tasks alone are insufficient for motor learning to occur and that instead training must involve problem solving and be goal-directed, task-specific, and challenging, but doable. Both of our protocols adhered to these principles. The research staff did not assist participants to move their limbs through the gait cycle, did not intervene to prevent losses of balance, and did not provide cues as to the “best” way to walk or perform a task. We instead allowed participants to develop their own movement strategies, learn from unexpected events, and problem-solve on their own to prevent similar occurrences. Both training groups walked in an active “intention-driven” environment with the same goal of maintaining their heart rate within the prescribed zone. Thus, all participants were provided with opportunities to make active adaptations to their walking pattern.

While groups improved CWS, average gains did not reach the minimal clinically important difference of 0.16 m/s [21]. However, 0.16 m/s is used as criteria for the subacute phase after stroke and there is no agreed upon clinically important difference for walking speed in the chronic phase [20]. One reason why we did not observe larger increases using these protocols may be that we allowed participants to walk at their comfortable speed during training. Studies with greater improvements in overground walking speed have instead used “maximum tolerable speed” [5, 19]. Additionally, our protocols were only 6 weeks and studies that noted larger improvements were generally longer in duration (e.g., 12 to 16 weeks) [9, 10]. Our observed speed improvements did, however, change the community ambulation status of many participants. We saw more limited community compared to household ambulators following training.

### Limitations

We acknowledge several limitations to the present study, including small sample sizes short of a priori power estimates and a large amount of data missing at follow-up. Additionally, our research question was designed to evaluate the effects of challenging mobility skills during treadmill training. We cannot address whether overground training plus mobility skills could have led to greater increases in overground walking speeds. The experimental design may also have been strengthened by an additional group that performed only challenging mobility skills without walking practice.

Regarding our lack of group differences in CWS, the HF + C group performed each mobility skill once per week for 10 min, which might have been insufficient dosing to yield greater improvements. While we ensured that each individual was challenged, we did not adapt skills to meet each individual’s unique requirements; thus, an individualized approach might have elicited greater gains. We must also consider that CWS might not be the best outcome measure to detect the types of improvements that the HF + C group may have experienced. All participants were in the chronic phase after stroke; however, treadmill training is shown to be effective in helping these individuals past “plateaus” in walking function [5]. While we had a wide range of time since stroke represented, our groups were well-balanced on this characteristic. Finally, we did not specifically exclude participants with minor musculoskeletal conditions commonly experienced poststroke (e.g., heel spur, plantar fasciitis, mild arthritis) so long as these conditions did not prevent engagement in training. While including these individuals yielded better ecological validity, it is possible that these comorbidities limited gains in walking function.

## Conclusions

The addition of challenging mobility skills to one of two comparable BWSTT protocols did not lead to greater improvements in walking and balance outcomes for individuals in the chronic phase poststroke. However, participants collectively improved CWS pre to post intervention resulting in changes in community ambulation status. These findings suggest that stroke survivors are capable of performing, and responding to, walking training that offers no assistance via handrails or therapist and may additionally incorporate essential skills related to competent walking function. In order to engender larger improvements in walking and balance outcomes poststroke future studies may consider incorporating challenges into walking interventions in combination with other promising strategies like task-specific training, individualized exercise prescription, and high-intensity training.

## Abbreviations

(ABC): Activities Specific Balance Confidence
(BBS): Berg Balance Scale
(BWSTT): Body-weight-support treadmill training
(CWS): Comfortable walk speed
(DGI): Dynamic Gait Index
(FWS): Fast walk speed
(GDS): Geriatric Depression Scale
(HF + C): Hands free + Challenge
(HF): Hands free
(SIS): Stroke Impact Scale
